# Cultivating Fluorescent Flowers with Highly Luminescent Carbon Dots Fabricated by a Double Passivation Method

**DOI:** 10.3390/nano7070176

**Published:** 2017-07-07

**Authors:** Shuai Han, Tao Chang, Haiping Zhao, Huanhuan Du, Shan Liu, Baoshuang Wu, Shenjun Qin

**Affiliations:** 1 College of Materials Science and Engineering, Hebei University of Engineering, Handan 056038, China; hansh04@163.com (S.H.); changt03@sina.com (T.C.); hebei123hh@sina.com (H.D.); ls10280924@sina.com (S.L.); hebeiwbs@163.com (B.W.); 2 Key Laboratory of Resource Exploration Research of Hebei Province, Hebei University of Engineering, Handan 056038, China; zhaohaiping609@163.com

**Keywords:** carbon dots, surface passivation, luminescence, Ca(OH)_2_, flowers

## Abstract

In this work, we present the fabrication of highly luminescent carbon dots (CDs) by a double passivation method with the assistance of Ca(OH)_2_. In the reaction process, Ca^2+^ protects the active functional groups from overconsumption during dehydration and carbonization, and the electron-withdrawing groups on the CD surface are converted to electron-donating groups by the hydroxyl ions. As a result, the fluorescence quantum yield of the CDs was found to increase with increasing Ca(OH)_2_ content in the reaction process. A blue-shift optical spectrum of the CDs was also found with increasing Ca(OH)_2_ content, which could be attributed to the increasing of the energy gaps for the CDs. The highly photoluminescent CDs obtained (quantum yield: 86%) were used to cultivate fluorescent carnations by a water culture method, while the results of fluorescence microscopy analysis indicated that the CDs had entered the plant tissue structure.

## 1. Introduction

As an emerging class of photoluminescent nanomaterials, carbon dots (CDs) have attracted considerable attention owing to their outstanding photostability, high biocompatibility, and tunable photoluminescence (PL) [1,2,3,4,5]. As this research field evolves, it is now widely recognized that the surface state of CDs significantly affects their fluorescence properties, and consequently their applicability in bioimaging, fluorescent inks, and so on [6,7,8,9,10]. Accordingly, it is especially important to enhance the PL by adjusting the surface chemistry of CDs [11,12]. Moreover, it is important to expand the applications of these types of materials, for example, to novel applications in biological technologies [13,14].

Citric acid, a surface passivation agent containing amino groups, has reportedly been used to prepare highly photoluminescent CDs by a hydrothermal method [15]. However, the active functional groups, such as amino and carboxyl groups, could be overconsumed during the reaction process, which might limit the PL quantum yield (QY) of the product [16]. On the other hand, the radiative recombination luminescence of the excitons could be absorbed by electron-withdrawing groups on the surface of the obtained CDs, such as carboxy and nitro groups, which might further limit the PL efficiency of the CDs [17]. Thus, it would be feasible to enhance the PL QY by choosing an appropriate agent that not only protects the active functional groups from overconsumption during the reaction process, but also reduces the number of electron-withdrawing groups on the CD surface.

With the rapid development of nanotechnology and biology, the applications of biocompatible nanomaterials become more and more attractive [18,19,20,21,22]. As a cross study of nanotechnology and phytobiology, the cultivation of function-specific plants using nanomaterials has become a hot research topic; for example, modified single-walled carbon nanotubes have been transported into plant leaves to monitor aromatic nitro compounds [23]. As a type of function-specific plant materials, fluorescent flowers, which emit fluorescence under ultraviolet (UV) light, are not only ornamental, but could also facilitate research in plant cell biology, environmental botany, and so on; consequently, they have been attracting increasing research interest [24]. To date, fluorescent flowers have been obtained mainly by transgenic technology [10]. However, this technology has disadvantages; for example, it is not cost effective and requires complex operations [25].

In this work, calcium hydroxide Ca(OH)_2_ was chosen as a representative passivating agent to produce CDs with much higher PL. In the reaction process, Ca^2+^ could protect the carboxyl groups of citric acid from overconsumption during dehydration and carbonization, and the electron-withdrawing groups could be converted to electron-donating groups (–OH) by the hydroxyl ions from Ca(OH)_2_. Thus, Ca^2+^ chelation in combination with hydroxyl group passivation, which we refer to as a double surface passivation strategy, was attempted. Then, the highly photoluminescent CDs obtained were used to cultivate fluorescent carnations by a water-culture method. To the best of our knowledge, this is the first study to cultivate fluorescent flowers using nanotechnology. Furthermore, the results of an insect attraction test showed that the fluorescent flowers are more attractive to insects than nonfluorescent ones in an outdoor environment, benefiting from the UV irradiation from solar spectrum.

## 2. Results

### 2.1. Physicochemical Characterization of CDs

**Figure 1:**
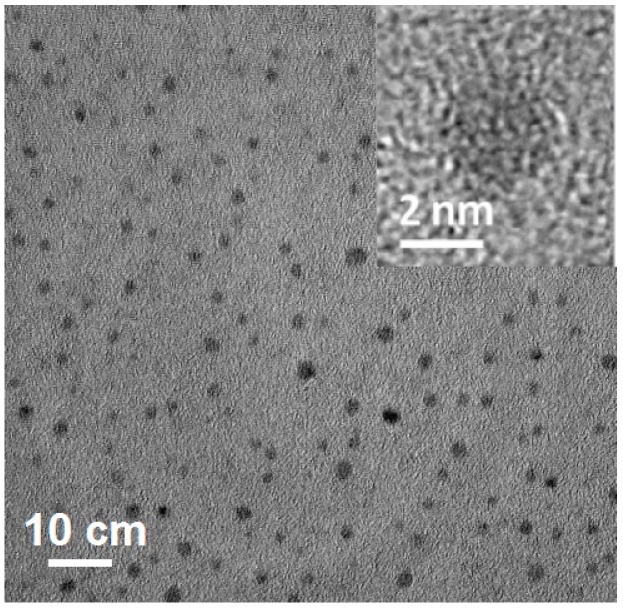
TEM image of Ca-2-CD (inset: HRTEM image).

Transmission electron microscope (TEM) was used to characterize the micromorphology of the three obtained CD samples. A TEM image (Figure 1) of Ca-2-CD indicates a uniform size (diameter: 2–5 nm) without apparent aggregation. A high-resolution TEM (HRTEM) image reveals that most of the Ca-2-CD particles are amorphous without any lattices [26,27]. Additional TEM images (Figure S1) show that the three CD samples (ECD, Ca-1-CD, and Ca-2-CD) had similar narrow size distributions and morphologies (average diameters: 3.3, 3.3, and 3.1 nm, respectively), indicating that adding Ca(OH)_2_ had no significant effect on the morphology of the CDs.

**Figure 2:**
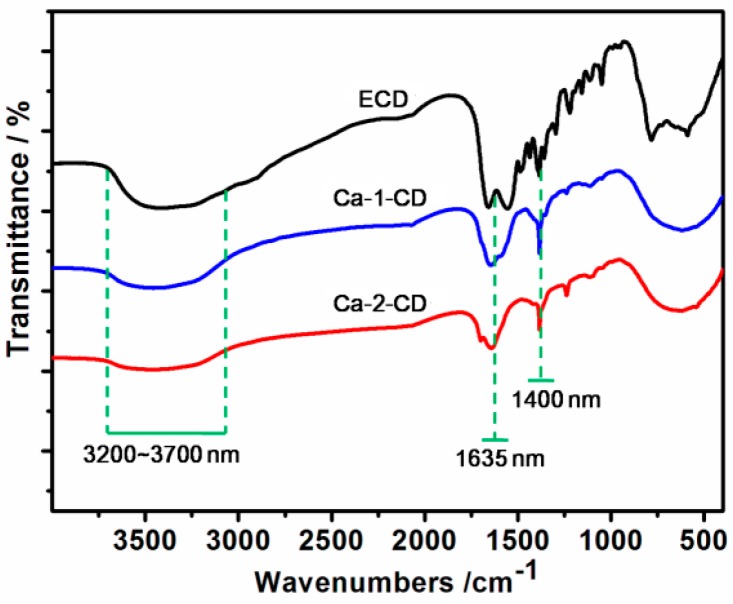
FTIR spectrum of the three CD samples.

**Figure 3:**
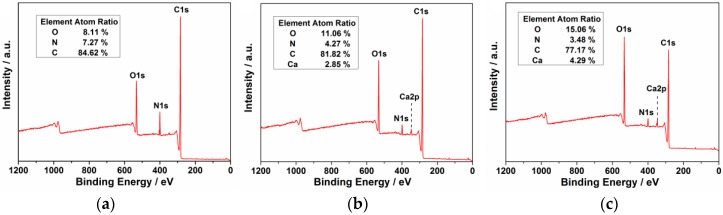
XPS spectra of the three CD samples. (**a**) ECD; (**b**) Ca-1-CD; (**c**) Ca-2-CD.

The FTIR results (Figure 2) revealed that all three samples showed O–H and N–H stretching at 3200–3700 cm^−1^, C=C stretching at 1420 cm^−1^, N–H bending at 1570 cm^−1^, and C=O stretching at 1635 cm^−1^ [28]. However, both Ca-1-CD and Ca-2-CD exhibited a similar prominent peak at 1400 cm^−1^, which is ascribed to the carboxylate anion (–COO^−^) and indicates successful anchoring of Ca^2+^ [29]. Moreover, it is worth noting that the intensity of O–H stretching (3200–3700 cm^−1^) and C=O stretching (1635 cm^−1^) gradually decrease as the amount of Ca(OH)_2_ increases, indicating conversion of the carboxyl groups to hydroxyl groups. XPS survey spectra provide a consistent result: the number of surface heteroatoms (Ca and O) increased as the amount of Ca(OH)_2_ increased (Figure 3a–c) [30,31]. Therefore, a possible mechanism for the formation of CDs passivated with Ca(OH)_2_ is proposed: as the amount of Ca(OH)_2_ increased, more Ca cations were anchored on the CD surface, which also converted the carboxyl groups to hydroxyl groups on the CD surface.

### 2.2. Photoluminescence Properties

**Figure 4:**
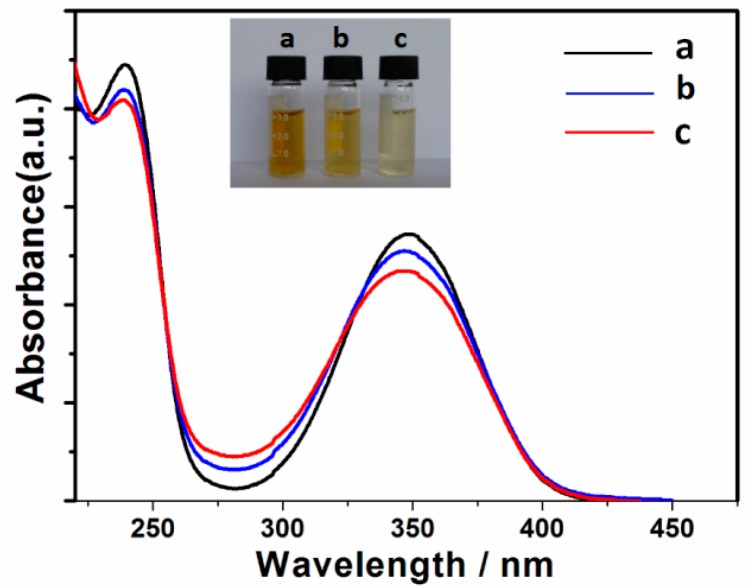
UV-Vis adsorption spectrum of the three CD samples in aqueous solution (0.1 μg·mL^−1^). (**a**) ECD; (**b**) Ca-1-CD; (**c**) Ca-2-CD.

The UV–Vis spectra of all three samples showed obvious similar absorption peaks in both the far-UV region (225–250 nm) and near-UV region (300–350 nm), which could be ascribed to the π–π* and *n*–π* transitions, and indicated the existence of aromatic structures in the CDs (Figure 4) [32]. However, the absorbance in the two regions decreased with an increasing amount of Ca(OH)_2_, and Ca-2-CD exhibited a slight blue shift (centered at 237 nm and 345 nm for the *π*–π* and n–π* transitions) compared with ECD (centered at 240 nm and 353 nm accordingly). We hypothesize that the carboxyl groups are auxochromes that can donate their lone-pair electrons to the antibonding orbitals of the *sp*^2^ carbon clusters, increasing the liquidity of the electron cloud in the conjugation system and thus decreasing their energy gaps and increasing the absorbance [33]. Consequently, as the number of carboxyl groups on the CD surface decreased, the absorbance in the optical spectrum decreased and a blue shift occurred. Furthermore, the energy gaps of the three CD samples were measured using cyclic voltammetry, which indeed showed a gradual increase with the increasing Ca(OH)_2_ content, and was consistent with our hypothesis (Figure S3).

**Figure 5:**
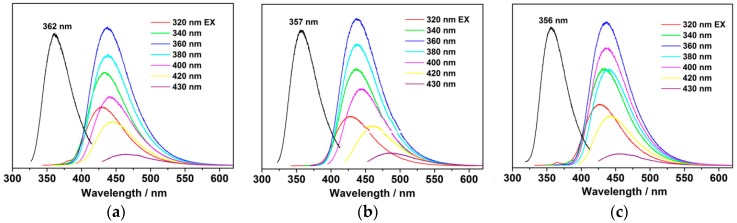
Excitation-dependent PL of the CD samples in aqueous solution. (**a**) ECD; (**b**) Ca-1-CD; (**c**) Ca-2-CD and the excitation spectrum monitored at the maximum emission peak (black line), (**a**) λ_em_ = 438 nm, λ_em_ = 434 nm, λ_em_ = 433 nm.

The smaller number of carboxyl groups on the CD surface also affected the PL behavior. As shown in Figure 5, the emission maximum is blue-shifted from 438 nm (ECD) to 433 nm (Ca-2-CD). The excitation maximum also exhibits corresponding blue shifts from 362 to 357 nm (monitored at the corresponding emission maximum). Clearly, new emission centers were generated as the amount of Ca(OH)_2_ increased. The QY, which is one of the most important function indicators, was also measured. Interestingly, the QY of the CDs was found to increase with increasing Ca(OH)_2_ content (Table S1). The QY of ECD reached 73.1%, whereas that of Ca-2-CD was further enhanced to as high as 86.0%, which is almost equal to that of organic dyes or semiconductor quantum dots [34]. Because of the extremely high PL, blue fluorescent emission from the aqueous solution of Ca-2-CD could be easily observed under natural light (Figure S2).

**Figure 6:**
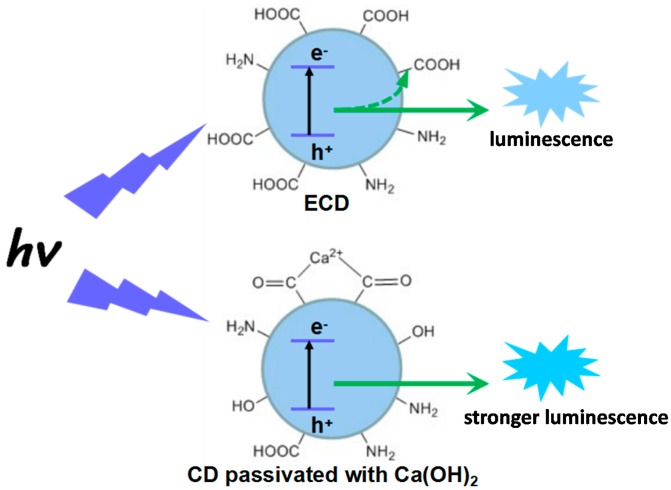
Schematic representation of the “double passivating strategy”.

The chelate rings of metallic chelates are known to possess excellent thermal stability, and it has been proven that the active functional groups can be protected by the chelating effect in the reaction process [16,35]. Thus, chelation of citric acid by Ca^2+^ can protect the carboxyl groups of citric acid from overconsumption during dehydration and carbonization. On the other hand, the radiative recombination luminescence of the excitons could be absorbed by the electron-withdrawing groups, such as carboxy and nitro groups, on the surface of the obtained CDs, which might limit the PL efficiency of the CDs. However, electron-donating groups, such as hydroxyl and amino groups, could produce a conjugation effect with the surface *sp*^2^ clusters of the CDs, which could further increase the transition probability between the lowest excited singlet state and the ground state of the surface *sp*^2^ clusters, thus enhancing the PL QY [36,37]. Thus, the QY of Ca-2-CD was further enhanced owing to the conversion of electron-withdrawing groups to electron-donating groups (–OH) by hydroxyl ions released from Ca(OH)_2_. Consequently, the double passivation strategy was found to produce the highly luminescent sample Ca-2-CD, as shown in Figure 6.

Time-resolved PL spectra of three CD samples were recorded (λ_ex_ = 360 nm) and fitted as two-component exponential decay functions, which all contain one fast and one slow components. The average of these components can be correlated to the energy transfer process among the *sp*^2^ clusters with different energy gaps. As shown in Figure S4 and Table S2, the average lifetimes of the three samples decreased with increasing Ca(OH)_2_ content in the reaction process. For the CD samples, fewer and fewer electron-withdrawing groups were attached on the CD surface with increasing Ca(OH)_2_ content, which made the energy gaps of the ECD be more and more simple, and subsequently, the frequency of the energy transfer process decreased. As the result, the average lifetime of the three samples decreased with increasing Ca(OH)_2_ content in the reaction process.

### 2.3. Biological Characteristics

**Figure 7:**
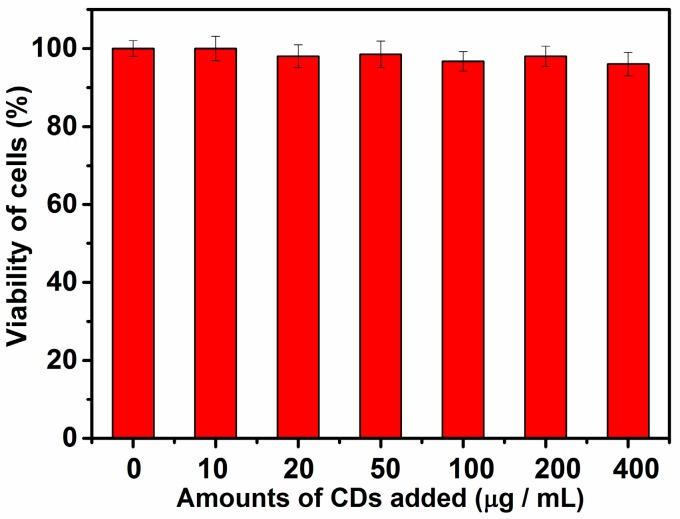
MTT assay for cytotoxicity values versus incubation concentrations (0–400 μg·mL^−1^) of Ca-2-CD.

The MTT assay method was used to evaluate the cytotoxicity of the as-prepared Ca-2-CD before application (Figure 7). It is noteworthy that the obtained CDs exhibit very little toxicity toward *HeLa* cells even at high doses (0.4 mg·mL^−1^) and after long incubation times (36 h); hence, they are safe and highly suitable for use in biological applications [38].

**Figure 8:**
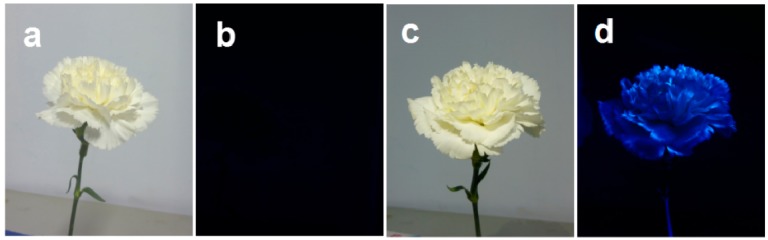
Photographs of carnation cultivated with D-I water (**a**,**b**) and Ca-2-CD solution (**c**,**d**); before (**a**,**c**) and after (**b**,**d**) UV irradiation.

Because of the high QY and low cytotoxicity, an exploratory experiment was performed to assess the potential application of Ca-2-CD for cultivating fluorescent flowers. As shown in Figure 8, cut carnations cultivated using DI water were observed to be nonfluorescent upon UV light irradiation; however, bright blue-light emission was observed under UV light irradiation from carnations cultivated using Ca-2-CD solution, which could be attributed to the CDs that had entered the plant. Interestingly, the margin of the petals was found to produce more intense fluorescence. It is thought that the CDs are small enough to be transported by the channels in the plant body when they are dispersed in water, and the water could be released through their leaves and petals by transpiration [39]. The margin is much thinner than other parts of the petals, making its air contact area much larger. Thus, transpiration would be much more intense at the margin, so the margin would accumulate much larger amounts of Ca-2-CD, ultimately producing stronger fluorescence. Furthermore, the longevity of the fluorescent cut flowers was observed comparing with that of the ordinary ones, which showed that there is no obvious influence of CDs-doping on the longevity of the cut flowers (Figure S5).

**Figure 9:**
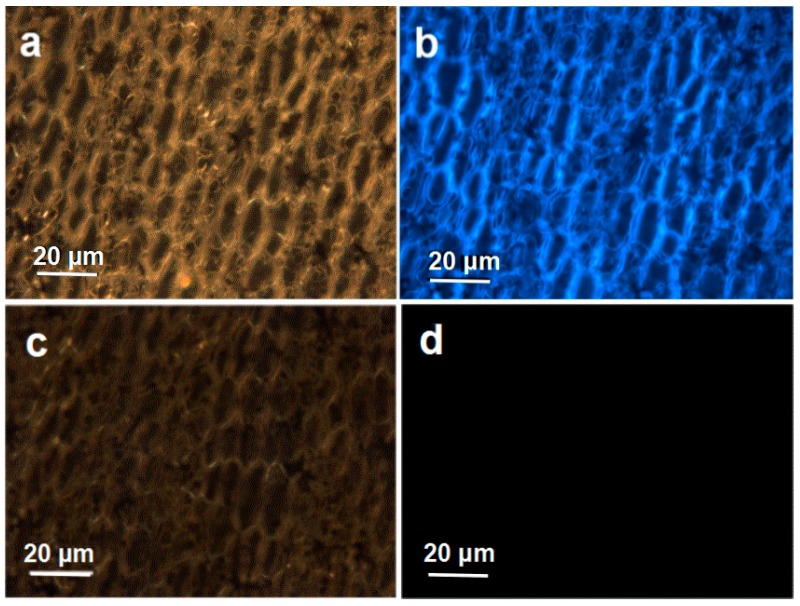
Fluorescence images of the petal tissue of fluorescent carnation at a micro level (**b**), Bright field transmission image (**a**); non-fluorescent carnation at a micro level. Bright field transmission image (**c**), Under UV light (**d**).

Fluorescence microscopy was used to investigate the distribution of Ca-2-CD in the plant body at the micro level. As illustrated in Figure 9c,d, petal tissue from the nonfluorescent flowers showed no fluorescence. However, an overlay of fluorescence and bright-field images of petal tissue from fluorescent carnations (Figure 9a,b) demonstrated that fluorescence could be observed in the tissue fluid and cells, indicating that the CDs had entered the plant tissue structure.

## 3. Methods

### 3.1. Materials

Citric acid and ethylenediamine were obtained from Aladin Ltd. (Shanghai, China). Carnations were purchased from Aimei Flowers Ltd. (Kunming, China). Deionized (DI) water was used for the experiments.

### 3.2. Measurements

Transmission electron microscopy (TEM) was performed on a JEOL-2010 instrument (JEOL, Tokyo, Japan) at 200 kV. Fourier transform infrared (FTIR) spectra were collected in the wavenumber range of 4000–400 cm^−1^ using a Nicolet 360 FTIR spectrometer (Nicolet, Madison, WI, USA). X-ray photoelectron spectroscopy (XPS) was performed using an ESCALAB 250 spectrometer (VG Scientific, London, England) with monochromatic Al Kα radiation (*hν* = 1486.6 eV), and the binding energy calibration was based on C 1s (284.6 eV). UV–vis absorption spectra were recorded on a PerkinElmer Lambda 950 spectrophotometer (PerkinElmer, Waltham, MA, USA). Excitation and emission spectra were collected using a Hitachi F-7000 fluorescence spectrophotometer (Hitachi, Tokyo, Japan). The QY of the CDs was measured at an excitation wavelength of 360 nm using quinine sulfate as a standard (QY = 54%) [6]. Confocal microscopy analysis was performed using an Olympus FluoView 500 laser scanning confocal microscope (Olympus, Tokyo, Japan), and the 3-(4,5-dimethylthiazol-2-yl)-2,5-diphenyltetrazolium bromide (MTT) assay of the obtained CDs was used to quantify the viability of *HeLa* cells [40].

### 3.3. Preparation of Carbon Dots

Citric acid (10 g), ethylenediamine (5 mL), and Ca(OH)_2_ (5.2 g) were dispersed in DI water (10 mL); the mixture was then transferred to an autoclave (100 mL) lined with poly(tetrafluoroethylene) (Teflon) and heated at 200 °C for 3 h. After the reaction, the reactors were cooled to room temperature naturally. The product, which was brown and transparent, was subjected to dialysis to obtain the CDs, denoted as Ca-2-CD. Simultaneously, to better understand the role of Ca(OH)_2_ in the formation of highly fluorescent CDs, parallel experiments were conducted with either 0 or 2.6 g of Ca(OH)_2_. The corresponding products are denoted as ECD and Ca-1-CD, respectively.

### 3.4. MTT Assay of Cell Viability

The cytotoxicity of Ca-2-CD was assessed by MTT assay. Briefly, approximately 1 × 10^4^ *HeLa* cells per well were seeded into a 96-well plate. After overnight culturing, the obtained Ca-2-CD was added to each well at concentrations ranging from 0 to 400 μg·mL^−1^ in Dulbecco’s modified Eagle’s medium. After incubation for 12 h, the metabolically active cells were then detected by adding MTT (5 mg·mL^−1^ in phosphate-buffered saline) for another 4 h, and the plates were read at 570 nm with a 630 nm reference. Then, the mean and standard deviation for triplicate wells were recorded.

### 3.5. Preparation of Fluorescent Flowers

Ca-2-CD solution was prepared with a concentration of 0.5 g·L^−1^. Cut carnations (flower diameter, 4–5 cm; stem length, 18–20 cm; cutting angle, 45°) were cultivated with the Ca-2-CD solution for 6 h at 25 °C. As a comparative test, other cut flowers were cultivated using DI water. To observe the fluorescence properties, the flowers were exposed to UV light with a wavelength of 365 nm below a 6 W UV lamp (10–20 cm apart) in a dark room.

### 3.6. The Longevity-Observing Tests of the Cut Flowers

Six carnations were put together as one bunch, then, the bunch of carnations was inserted in the Ca-2-CD solution with a concentration of 0.5 g·L^−1^. During the cultivation, the solution was freshly changed every 24 h. The longevity of the fluorescent cut flowers was observed and recorded. As a comparative test, the other bunch of cut flowers was cultivated using DI water. The longevity-observing tests were arranged in four groups to make analysis.

### 3.7. Confocal Microscopy

Confocal microscopy analysis was performed using an Olympus BX51 fluorescence microscope (Olympus, Tokyo, Japan). Briefly, a small piece of skin was torn from the petal of a fluorescent carnation using tweezers and quickly fixed on a slide for inspection and bioimaging using the fluorescence microscope.

## 4. Conclusions

In summary, a double passivation strategy was developed to fabricate highly luminescent CDs with the assistance of Ca(OH)_2_. In the reaction process, Ca^2+^ could protect the active functional groups from overconsumption during dehydration and carbonization, and electron-withdrawing groups could be converted to electron-donating groups (–OH) on the CD surface by hydroxyl ions. Then, the highly photoluminescent CDs obtained were used to cultivate fluorescent carnations by a water-culture method. Further, the results of an insect attraction test showed that the fluorescent flowers are more attractive to insects than non-fluorescent ones in an outdoor environment. Our work provides a new method of preparing highly luminescent CDs and is expected to afford a new way to obtain function-specific plants.

## Supplementary Materials

The following are available online at http://www.mdpi.com/2079-4991/7/7/176/s1. Figure S1: TEM images of ECD (a), Ca-1-CD (b), Ca-2-CD (c), Figure S2: The Ca-2-CD aqueous solution under natural light irradiation (0.1 μg·mg^−1^), Figure S3: The cyclic voltammogram of the ECD (a), Ca-1-CD (b), Ca-2-CD (c) and 0.1 mol/L KCl aqueous solution, Figure S4: Luminescence decay curve of the three CD samples recorded at room temperature in aqueous solution (a. ECD, b. Ca-1-CD, c. Ca-2-CD), Figure S5: The results of the longevity-observing tests (left, the longevity of ordinary carnations; right, the longevity of fluorescent carnations), Table S1: The QY results of the three CD samples in aqueous solution, Table S2: The lifetimes (*τ*) and the average lifetimes (<*τ*>) of the three CD samples.
