# Supplementary material for: Cultivating Fluorescent Flowers with Highly Luminescent Carbon Dots Fabricated by a Double Passivation Method
Source: Nanomaterials (Basel). 2017 Jul 7;7(7):176. doi: 10.3390/nano7070176 (PMC5535242; doi:10.3390/nano7070176)
Supplement: Supplementary file 1 [file nanomaterials-07-00176-s001.pdf]

Supplementary Nanomaterials: Cultivating Fluorescent Flowers with Highly Luminescent Carbon Dots Fabricated by A Double Passivation Method

Shuai Han [1,2], Tao Chang [1], Haiping Zhao [2], Huanhuan Du [1], Shan Liu [1], Baoshuang Wu [1] and Shenjun Qin [2,*]

[1] College of Materials Science and Engineering, Hebei University of Engineering, Handan 056038, China; hansh04@163.com (S.H.); changt03@sina.com (T.C.); hebei123hh@sina.com (H.D.); ls10280924@sina.com (S.L.); hebeiwbs@163.com (B.W.)

[2] Key Laboratory of Resource Exploration Research of Hebei Province, Hebei University of Engineering, Handan 056038, China; hansh04@163.com (S.H.); zhaohaiping609@163.com

* Correspondence: qinsj528@hebeu.edu.cn (S.Q.); Tel.: +86-0310-857-7902

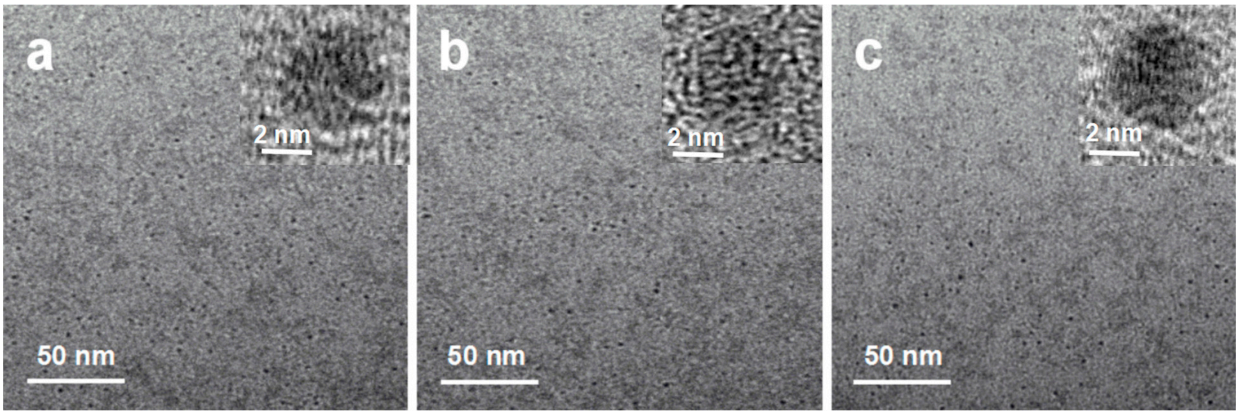

Figure S1. TEM images of ECD (a), Ca-1-CD (b), Ca-2-CD (c).

Table S1. The QY results of the three CD samples in aqueous solution.

	ECD	Ca-1-CD	Ca-2-CD
QY(%)	73.1	81.3	86.1

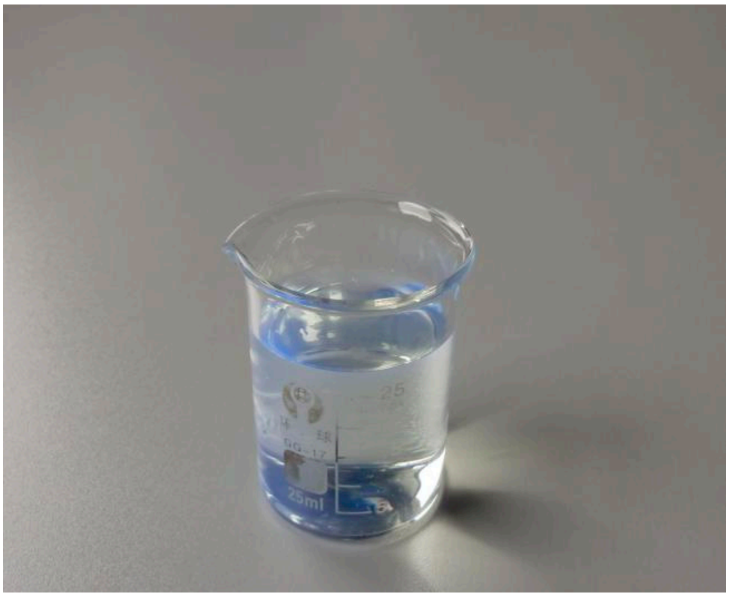

Figure S2. The Ca-2-CD aqueous solution under natural light irradiation (0.1 μg·mg^{-1}).

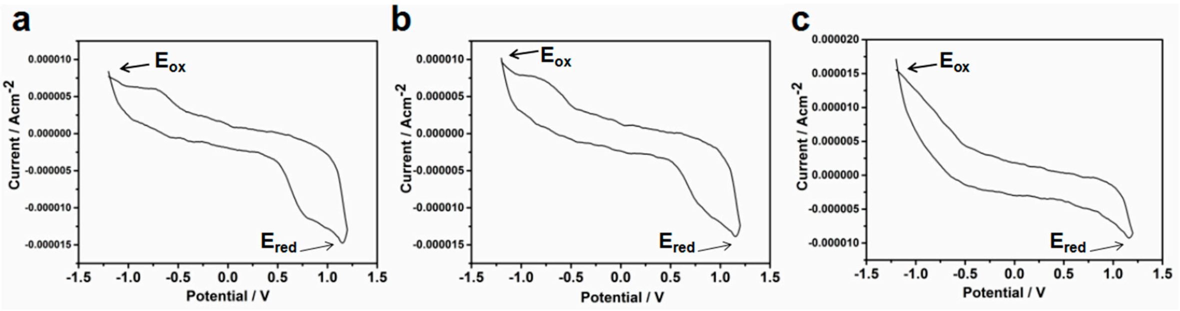

Figure S3. The cyclic voltammogram of the ECD (a), Ca-1-CD (b), Ca-2-CD (c) and 0.1 mol/L KCl aqueous solution (the scan rate: 30 mV/s).

Data notes:

To estimate their HOMO and LUMO energy levels, cyclic voltammetry (CV) was carried out by using a standard three-electrode system, which consisted of glassy carbon electrode as the working electrode, a platinum wire as the counter electrode, and calomel electrode as the reference electrode. CV was recorded in DI-water containing CMCD and 0.1 M KCl as the supporting electrolyte. The HOMO and LUMO energy levels in eV of CMCD were calculated according to the following equations:

$$E(\mathrm{HOMO}) = -\mathrm{e}(E_{\mathrm{ox}} + 4.4)\ (\mathrm{eV}) \quad (1)$$

$$E(\mathrm{LUMO}) = -\mathrm{e}(E_{\mathrm{red}} + 4.4)(\mathrm{eV}) \quad (2)$$

$$E_{\mathrm{g}} = -\mathrm{e}\Delta E \quad (3)$$

$$\Delta E = E_{\mathrm{ox}} - E_{\mathrm{red}} \quad (4)$$

where E_{ox} and E_{red} are the onset of oxidation and reduction potential, which are the potentials corresponding to the the maximum forward current and the backward current. E_{g} is the energy gap, respectively.[S5]

Finally, we could calculate the energy gaps listed as below:

E_{ECD} = 3.57 eV, $E_{\mathrm{Ca\text{-}1\text{-}CD}}$ = 3.60 eV, $E_{\mathrm{Ca\text{-}2\text{-}CD}}$ = 3.62 eV

where E_{ECD}, $E_{\mathrm{Ca\text{-}1\text{-}CD}}$ and $E_{\mathrm{Ca\text{-}2\text{-}CD}}$ are the energy gaps of ECD, Ca-1-CD and Ca-2-CD, respectively.

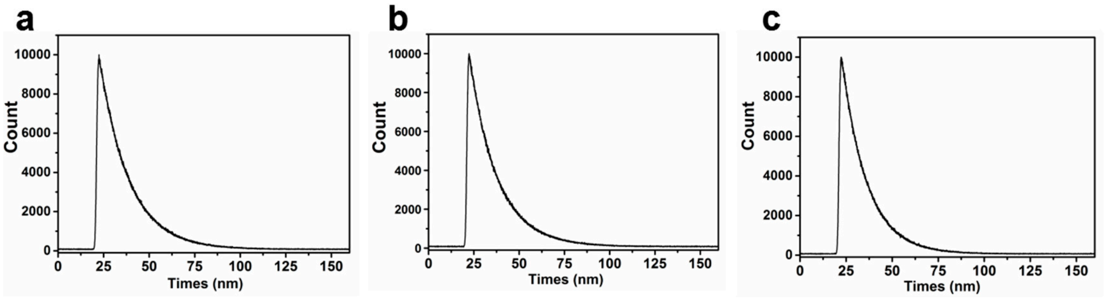

Figure S4. Luminescence decay curve of the three CD samples recorded at room temperature in aqueous solution (a. ECD , b. Ca-1-CD, c.Ca-2-CD).

Data notes:

The emission decay curve was monitored under the excitation wavelength at 360 nm, and two exponents were shown for the three curves (Table S2). The average lifetime <τ> is estimated by the following equation:

$$\langle\tau\rangle = \frac{\sum A_i \tau_i^2}{\sum A_i \tau_i}$$

where A_i is the preexponential factor related to the statistical weights of each exponential and τ_i represent the lifetimes of each exponential decay. The lifetimes of the CD samples are shown below:

Table S2. The lifetimes (τ) and the average lifetimes (<τ>) of the three CD samples.

	**ECD**	**Ca-1-CD**	**Ca-2-CD**

τ_1	3.86 ns (1.6%)	3.41 ns (1.0%)	3.2 ns (1.1%)
τ_2	15.42 ns (98.4%)	15.01 ns (99.0%)	14.5 ns (98.9%)
$<\tau>$	15.23 ns	14.89 ns	14.7 ns

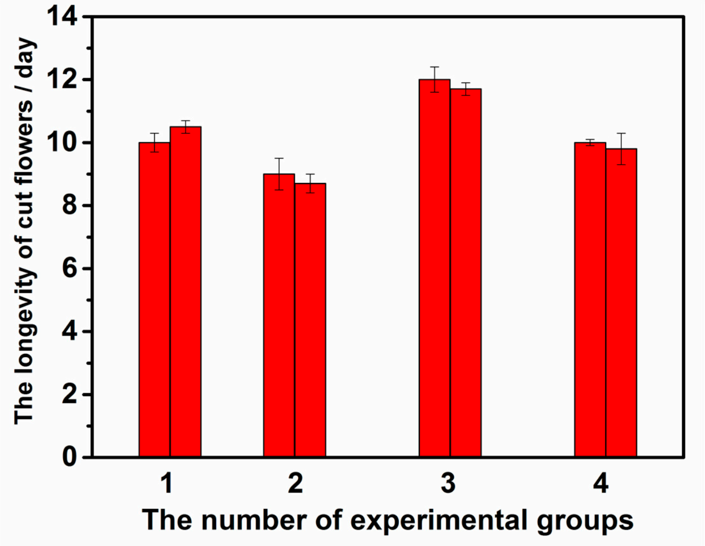

Figure S5. The results of the longevity-observing tests (left,the longevity of ordinary carnations; right, the longevity of fluorescent carnations).
